# The natural history and genotype–phenotype correlations of *TMPRSS3* hearing loss: an international, multi-center, cohort analysis

**DOI:** 10.1007/s00439-024-02648-3

**Published:** 2024-04-30

**Authors:** Brett M. Colbert, Cris Lanting, Molly Smeal, Susan Blanton, Derek M. Dykxhoorn, Pei-Ciao Tang, Richard L. Getchell, Hedwig Velde, Mirthe Fehrmann, Ryan Thorpe, Prem Chapagain, Heidy Elkhaligy, Hannie Kremer, Helger Yntema, Lonneke Haer-Wigman, Shelby Redfield, Tieqi Sun, Saskia Bruijn, Astrid Plomp, Thadé Goderie, Jiddeke van de Kamp, Rolien H. Free, Jolien Klein Wassink-Ruiter, Josine Widdershoven, Els Vanhoutte, Liselotte Rotteveel, Marjolein Kriek, Marieke van Dooren, Lies Hoefsloot, Heriette H. W. de Gier, DOOFNL Consortium, M. F. van Dooren, S. G. Kant, H. H. W. de Gier, E. H. Hoefsloot, M. P. van der Schroeff, L. J. C. Rotteveel, F. G. Ropers, M. Kriek, E. Aten, J. C. C. Widdershoven, J. R. Hof, K. Hellingman, V. Vernimmen, H. Kremer, R. J. E. Pennings, I. Feenstra, C. P. Lanting, H. G. Yntema, F. L. J. Cals, L. Haer-Wigman, R. H. Free, J. S. Klein Wassink-Ruiter, A. L. Smit, M. J. van den Boogaard, A. M. A. Lachmeier, J. J. Smits, F. A. Ebbens, S. M. Maas, A. Plomp, T. P. M. Goderie, P. Merkus, J. van de Kamp, Amanda Schaefer, Diana Kolbe, Hela Azaiez, Grace Rabie, Armal Aburayyan, Mariana Kawas, Moien Kanaan, Jourdan Holder, Shin-ichi Usami, Zhengyi Chen, Pu Dai, Jeffrey Holt, Rick Nelson, Byung Yoon Choi, Eliot Shearer, Richard J. H. Smith, Ronald Pennings, Xue Zhong Liu

**Affiliations:** 1 Department of Otolaryngology, University of Miami Miller School of Medicine, 1120 NW 14th Street, 5th Floor, Miami, FL 33136 USA; 2 Medical Scientist Training Program, University of Miami Miller School of Medicine, Miami, USA; 3 Dr. John T Macdonald Foundation Department of Human Genetics, University of Miami Miller School of Medicine, Miami, USA; 4 Department of Otorhinolaryngology, Radboud University Medical Center, Nijmegen, The Netherlands; 5 Department of Otolaryngology, University of Iowa, Iowa City, USA; 6 Boston Children’s Hospital, Boston, USA; 7 Amsterdam University Medical Center, Amsterdam, The Netherlands; 8 Groningen University Medical Center, Groningen, The Netherlands; 9 Maastricht University Medical Center, Maastricht, The Netherlands; 10 Leiden University Medical Center, Leiden, The Netherlands; 11 Erasmus Medical Center, Rotterdam, The Netherlands; 12 Hereditary Research Laboratory and Department of Life Sciences, Bethlehem University, Bethlehem, Palestine; 13 Vanderbilt University, Nashville, USA; 14 Shinshu University School of Medicine, Matsumoto, Japan; 15 Eaton-Peabody Laboratory, Massachusetts Eye and Ear Infirmary and Department of Otolaryngology-Head and Neck Surgery, Harvard Medical School, Boston, USA; 16 PLA General Hospital, Beijing, China; 17 Department of Otolaryngology, Indiana University School of Medicine, Indianapolis, USA; 18 Seoul National University Bundang Hospital, Seongnam, South Korea; 19 Department of Physics and Biomolecular Sciences Institute, Florida International University, Miami, USA; 20 University of Washington, Seattle, USA

## Abstract

*TMPRSS3*-related hearing loss presents challenges in correlating genotypic variants with clinical phenotypes due to the small sample sizes of previous studies. We conducted a cross-sectional genomics study coupled with retrospective clinical phenotype analysis on 127 individuals. These individuals were from 16 academic medical centers across 6 countries. Key findings revealed 47 unique *TMPRSS3* variants with significant differences in hearing thresholds between those with missense variants versus those with loss-of-function genotypes. The hearing loss progression rate for the DFNB8 subtype was 0.3 dB/year. Post-cochlear implantation, an average word recognition score of 76% was observed. Of the 51 individuals with two missense variants, 10 had DFNB10 with profound hearing loss. These 10 all had at least one of 4 TMPRSS3 variants predicted by computational modeling to be damaging to TMPRSS3 structure and function. To our knowledge, this is the largest study of TMPRSS3 genotype–phenotype correlations. We find significant differences in hearing thresholds, hearing loss progression, and age of presentation, by TMPRSS3 genotype and protein domain affected. Most individuals with TMPRSS3 variants perform well on speech recognition tests after cochlear implant, however increased age at implant is associated with worse outcomes. These findings provide insight for genetic counseling and the on-going design of novel therapeutic approaches.

**Supplementary Information:**

The online version contains supplementary material available at 10.1007/s00439-024-02648-3.

## Introduction

Hearing loss is the most common sensory disorder. The World Health Organization estimates 20% of the global population has some degree of hearing loss, with more than 430 million people experiencing disabling hearing loss (WHO 2023). Hearing loss can be caused by various factors, including aging, ototoxic drugs, traumatic injury, infection, and genetic factors, with over 120 hearing loss-associated genes identified thus far (Van Camp 2023).

**Fig. 1:**
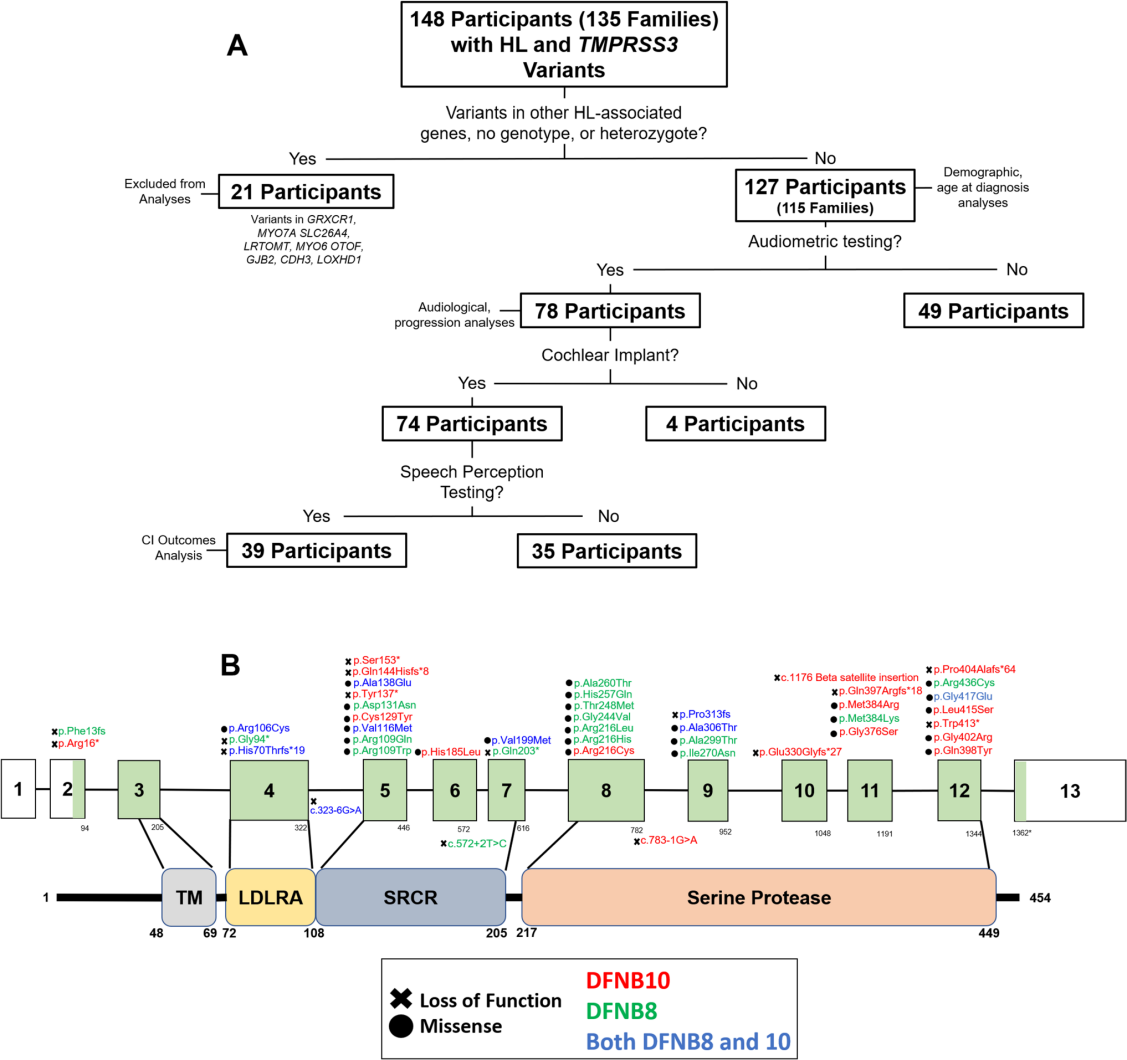
**A** Participants included in each analysis. We received data about 148 individuals in 135 families from 16 centers in 6 countries. 21 had variants in other known HL-related genes, had no TMPRSS3 genotype reported, or were heterozygous for TMPRSS3 mutations and were excluded from analysis. 78 participants had audiometric testing reported. 74 participants had cochlear implants and 39 of these reported the results of speech perception testing after cochlear implantation. **B** Locations of the *TMPRSS3* variants in the data set. There were 48 unique *TMPRSS3* variants in the data set. Variants included nonsense, missense, splice site, and indel frameshift variants. Variants in red were associated with DFNB10 and green with DFNB8. Variants in blue were associated with both DFNB8 and DFNB10. The black ‘X’ indicates loss of function variants and the black circle indicates missense

The Transmembrane Protease, Serine 3 (*TMPRSS3*) gene is predicted to affect 1–12% of individuals with genetic hearing loss, depending on ethnic background (Gao et al. 2017; Scott et al. 2001; Wattenhofer et al. 2002, 2005). *TMPRSS3* encodes a serine protease that is expressed in many cell types of the inner ear, including the sensory hair cells, supporting cells, and spindle/root cells, with limited expression in the type II spiral ganglion neurons (Chen et al. 2022; Guipponi et al. 2002). TMPRSS3 has four domains: a transmembrane domain (TM), a low-density lipoprotein receptor A domain (LDLRA), a scavenger receptor cytosine-rich domain (SRCR), and a serine protease domain (Figs. 1B, 5A). Although TMPRSS3 has been shown to activate epithelial sodium channels (ENaC) (Guipponi et al. 2002) and impact the level of the calcium-activated potassium (BK) channel KCNMA1 (Molina et al. 2013; Tang et al. 2019), the exact role of TMPRSS3 in hearing—and by association in hearing loss—is poorly understood.

Clinically, genetic variants in *TMPRSS3* cause non-syndromic, autosomal recessive deafness 8 and 10 (DFNB8 and DFNB10) (Bonne-Tamir et al. 1996; Nisenbaum et al. 2023; Scott et al. 2001; Veske et al. 1996). DFNB8 is characterized by childhood-onset, sloping hearing loss: mild-to-moderate at the low frequencies and severe-to-profound at the high frequencies (Veske et al. 1996). Conversely, DFNB10 shows congenital, severe-to-profound hearing loss at all frequencies (Bonne-Tamir et al. 1996). The simplicity of this dichotomy may not capture the spectrum of effects that depend on variant type, domain affected, and the combinations of variants in any given individual.

Although *TMPRSS3* variants have been reported in many populations worldwide, the genetic epidemiology and genotype–phenotype correlations of these variants remain poorly understood. This is largely due to the relatively small sample sizes of previous studies. In this study, we aimed to investigate the clinical and genetic features of *TMPRSS3*-related hearing loss by collecting data from a large, international cohort of affected individuals. We collected clinical genetic testing, serial audiograms, age at hearing loss diagnosis, hearing-assistive device status, speech perception testing after cochlear implantation, and hearing loss progression over time from 148 participants recruited from 16 centers in 6 countries. We analyzed the genetic and clinical features of the participants to explore the genotype-to-phenotype correlations of *TMPRSS3* variants, specifically audiological patterns, hearing loss progression over time, cochlear implant outcomes, and predictive protein structure modeling of *TMPRSS3* variants for use in future experimentation. Our results provide new insights into the genetic basis of *TMPRSS3*-related hearing loss, which has implications for genetic counseling and the timing of targeted therapies currently in development.

## Methods

Our cross-sectional study design (genetics) and retrospective cohort analysis (clinical phenotypes) followed the Strengthening the Reporting of Observational Studies in Epidemiology (STROBE) guidelines (Von Elm et al. 2007). Data were collected under protocols approved by the institutional review boards of the respective institutions. All data were provided to the University of Miami with alphanumeric codes and no identifiable information. Analysis was performed under protocols approved by the institutional review board at the University of Miami (IRB protocol: 20010415).

### Participants and genetics

Participants were recruited retrospectively from 16 centers across 6 countries (eFigure 1). A request for data that included a data collection sheet with the variables to report for each participant was sent to each center. Inclusion criteria were presentation with bilateral, sensorineural hearing loss and a clinical genetic report with variants in *TMPRSS3.* We received data on 148 individuals who met the inclusion criteria. Individuals with genetic variants in other known HL-associated genes, no genotype reported, or were heterozygous for a single variant in *TMPRSS3* were excluded (21 individuals). Demographics of the 127 individuals included in the analyses are available in eTable 1. Seventy-eight participants had audiograms available. Seventy-four participants had cochlear implants and word recognition scores were available for 39 of these (Fig. 1A).

### Genetics

Individuals were grouped by family for allele frequency analysis and only one individual per family was included in subsequent audiological analyses (see below). The 127 individuals were from 115 families.

Clinical genetic testing included GeneDx exome panel (Gaithersburg, MD), OtoSCOPE (Iowa City, IA), and whole exome sequencing. For further analyses, participants were grouped by DFNB diagnosis (8 vs. 10; based on age at hearing loss onset and severity/shape of hearing loss by audiogram) and genotypic categories. Each variant was classified as either missense or loss of function. Loss of function includes insertion/deletion (indel)-frameshift, nonsense, and splice site variants. Genotype categories were based on the combinations of the variant types: missense/missense (M/M), missense/loss of function (M/LoF), and loss of function/loss of function (LoF/LoF).

### Audiology

Audiological testing results were collected for 78 individuals. Hearing thresholds were determined for frequencies between 125 Hz and 8 kHz by pure tone audiometry. Tests were performed at each of the centers, and original, deidentified test reports were collected. All tests were interpreted by one audiologist (M.S.). At least four frequencies had to be reported for a test to be included in the analysis. Missing values were imputed by interpolation between the reported frequencies.

Since many subjects had multiple audiograms, we used linear mixed models (LMMs). LMMs are well suited for examining repeated measures and capturing shared variance within subjects while accounting for between-subject differences. For this, we modelled the effects of mutations in three domains (LDLRA, SRCR, or Serine Protease) on the audiogram, represented by the thresholds for three frequency bands (low, mid, and high). We examined a total of 4 subjects in the LDLRA domain, 14 in the SRCR domain, and 17 in the Serine Protease domain. Additionally, we investigated the potential influence of age as a factor and explored whether the domain itself had a differential effect on the shape of the audiogram. The LME4 package (version 1.1-31) in RStudio (version 2023.03.0 + 446) was used to fit the model. Subject ID was treated as a random factor, while the other factors and interactions were considered fixed factors. The significance of the factors was evaluated by examining effect sizes, *F*- and *p*-values, and the proportion of explained variance (R-squared).

Pure tone averages (PTA) were calculated for individuals with DFNB8 by averaging the thresholds of 500, 1000, 2000, and 4000 Hz for a given audiogram. Hearing loss progression in DFNB8 progression was determined by plotting PTA by age at test (Fig. 3A) and fitting a linear regression. Thresholds for frequencies 250–8000 Hz were also plotted by age and grouped by genotype category for linear regression (Fig. 3B).

Speech perception after cochlear implantation was determined by word recognition score (WRS) using the implant and reported as a percentage of words correctly identified. WRS tests used were consonant–vowel-consonant (CVC) (Causey et al. 1984), HINT (Nilsson et al. 1994), AzBio (Spahr et al. 2012), W-22 (Auditec, St. Louis, Missouri), and the CVC NVA (Dutch language version) (Causey et al. 1984), all presented at 65 dB SPL. Age at implantation was included as a covariate.

### Structural analyses of missense variants

The structure of the canonical TMPRSS3 protein (UniProt id: P57727) was obtained from AlphaFold2 (Jumper et al. 2021; Varadi et al. 2022). The missense mutations of TMPRSS3 were analyzed for their potential effect on protein stability, structure, and function using various web servers, including mCSM (Pires et al. 2014), SIFT (Sim et al. 2012), missense 3D (Ittisoponpisan et al. 2019; Khanna et al. 2021), and polyphen2 (Adzhubei et al. 2013, 2010). Selected mutations were visualized using ChimeraX (Pettersen et al. 2021). The model of the lipid bilayer containing the TMPRSS3 was built using the Charm-gui membrane builder (Jo et al. 2008; Lee et al. 2018; Wu et al. 2014) and visualized with ChimeraX.

### Statistical analysis

**Fig. 2:**
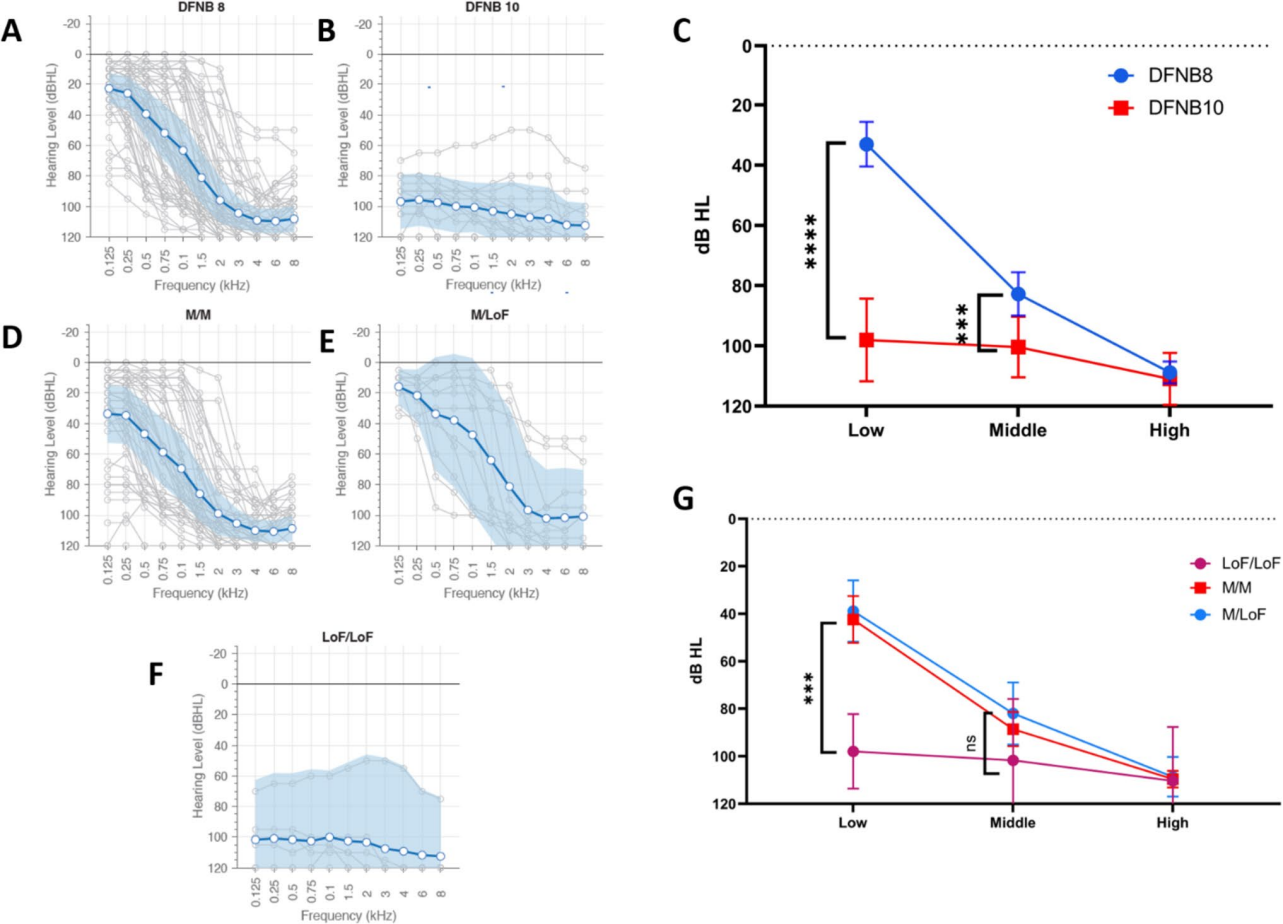
Audiometric thresholds by DFNB8/10 and by genotypic categories. Audiograms were collected for 78 individuals. Grey lines are a family (when a family had more than one audiogram, one was chosen at random for **A**, **B** and **D**–**F**; **C** and **G** are representative of all audiograms [see methods]), blue line is the mean at each frequency, and the blue shading is the 95% CI for DFNB8 (**A**), DFNB10 (**B**), and the 3 genotypic categories, M/M (**D**), M/LoF (**E**), LoF/LoF (**F**). **C** The mean thresholds of DFNB8 and DFNB10 differ at low (250 + 500 Hz) and middle (1 + 2 kHz) frequencies (student’s *t* test; error, 95% CI). **G** The mean thresholds of the 3 genotypes at low, middle, and high frequencies (one-way ANOVA with Tukey’s post-hoc comparison). ***p < 0.0005; **p < 0.005; *p < 0.05, ns = not significant

Statistical analysis was performed using a two-tailed student t-test, analysis of variance (ANOVA) with Tukey’s comparison, and linear regression as appropriate and indicated in the figures. For the audiogram analyses (Fig. 2), the better hearing ear at each frequency was used to generate a single audiogram per individual per time point (Taylor et al. 2013). Means were calculated at low (250 + 500 Hz), middle (1 + 2 kHz), and high frequencies (4 + 8 kHz), and ANOVA with Tukey’s comparison was performed to obtain a *p* value. To account for sampling bias, a Monte Carlo approach was used: the process was repeated 10,000 times, sampling a different set of audiograms each time with one audiogram per family (Thorpe et al. 2022; Walls et al. 2020). The average *p* value across the 10,000 runs was calculated and reported. Age at each audiogram was included as a covariate.

All data were normally distributed (except age at hearing loss diagnosis), and parametric tests were used. Alpha was set to 0.05. Means with 95% confidence intervals are reported.

## Results

### TMPRSS3 variants

There were 127 participants in 115 families. Among the 115 families included in the analysis, the distribution of *TMPRSS3* genotypes were as follows: 71 families (62%) had M/M genotypes, 29 families (25%) had M/LoF genotypes, and 15 individuals (13%) had LoF/LoF genotypes (Fig. 1A). In our dataset, we identified a total of 47 unique TMPRSS3 variants (Fig. 1B). These variants comprised 29 missense variants (62%), 9 indel-frameshift variants (19%), 6 nonsense variants (13%), 1 beta satellite insertion variant (2%), and 3 splice site variants (5%). These variants occurred across all *TMPRSS3* exons and in all 4 TMPRSS3 protein domains (Fig. 1B). There were 3 variants (< 1%) in the LDL-receptor domain (LDLRA), 12 (30%) in the scavenger receptor cytosine rich domain (SRCR), and 25 (60%) in the serine protease domain (eTable 6). Missense variants at the beginning of the serine protease domain were more often associated with DFNB8, while missense variants at the end of the serine protease domain were associated with DFNB10 (Fig. 1B). There were 2 (3%) loss of function variants (one nonsense and one indel-frameshift) that occurred before the TM domain and are predicted to cause a loss of all functional domains (Fig. 1B). We identified ten novel *TMPRSS3* variants (eTable 6). The most common variants by allele frequency were 24% c.916G>A (p.Ala306Thr), 18% c.413C>A (p.Ala138Glu), 11% c.208delC (p.His70Thrfs*19), and 4% c.1190delA (p.Gln397Argfs*18). A complete list of variants with allele frequencies is available in eTable 6.

### Age at hearing loss diagnosis

Due to the potential for delayed diagnosis, we could not reliably determine the age at hearing loss onset for each individual in the study. In lieu of this information, we recorded age at presentation by self-report and hearing loss diagnosis. Individuals with either M/M (13.2 years; 95% CI 10.0–16.5 years) or M/LoF (8.41 years; 95% CI 3.2–13.6 years) genotypes have a later age of diagnosis than those with an LoF/LoF genotype (0.1 years; 95% CI 0–0.2 years) (one-way ANOVA with Tukey’s post hoc comparisons; M/M to LoF/LoF, *p* = 0.006; M/LoF to LoF/LoF, *p* = 0.05; M/M to M/LOF, *p* = 0.73; eFigure 2B).

### Audiometry findings by DFNB and genotypic groups

Individuals with a DFNB8 diagnosis have a sloping audiogram with mild-to-moderate hearing loss at the low (33.0 dB HL) and middle (82.7 dB HL) frequencies and profound hearing loss (108.8 dB HL) at higher frequencies (Fig. 2A). Individuals with a DFNB10 diagnosis display a slightly sloping audiogram (Fig. 2B), but all thresholds show profound hearing loss (low, 78.5 dB HL; middle, 100.3 dB HL; high, 111.0 dB HL). DFNB10 shows significantly higher hearing thresholds than DFNB8 at low and middle frequencies (Fig. 2C; low, *p* < 0.00001; middle, *p* = 0.004; high, *p* = 0.7; students *t* test).

M/M (Fig. 2D; low 42.3 dB HL; middle 88.5 dB HL; high 109.6 dB HL) and M/LoF (Fig. 2E; low 38.8 dB HL, middle 82.0 dB HL, high 108.6 dB HL) display sloping audiograms. There are 10 M/M and 8 M/LoF individuals with DFNB10. These 18 individuals show flat audiograms with profound hearing loss at each frequency measured, contrary to the mean of these genotype groups. LoF/LoF displays a flat, profound hearing loss (Fig. 2F; low 97.9 dB HL, middle 101.7 dB HL, and high 110.3 dB HL). LoF/ LoF genotypes have significantly higher thresholds at low frequencies (*p* = 0.0005, one-way ANOVA with Tukey’s comparison), but not at middle or high frequencies (Fig. 2H).

### Audiometry by protein domain and individual variants

Audiograms were grouped by protein domain for individuals with two different variants in the same domain. Analysis of the threshold as a function of frequency (PTA low, mid and high), domain, and age using a linear mixed model and ANOVA showed that the domain affected is significantly impacting threshold (*F*(2, 92) = 6.0108, *p* = 0.00352). Moreover, the PTA across the frequency domains is highly significant (*F*(2, 92) = 47.0201, *p* < 0.001). Importantly, this is not a function of age (*F*(1, 92) = 0.0000, *p* = 0.99604). The interaction of domain and frequency shows a sloping audiogram for the serine protease domain and the SCRC domain and a flat audiogram for the LDLRA domain and trends towards significance (*F*(4, 92) = 2.3277, *p* = 0.06199).

Variants in the SCRC domain (*n* = 17; eFigure 3A; low 32.5 dB HL; middle 77.3 dB HL; high 102.7 dB HL) and serine protease domain (*n* = 14; eFigure 3B; low 47.5 dB HL; middle 94.2 dB HL; high 113.1 dB HL) had sloping audiograms and the LDLRA domain variants had a flat audiogram (*n* = 4; eFigure 3C; low 98.1 dB HL; middle 100.0 dB HL; high 103.9 dB HL).

Audiograms were grouped by individual variants for those who were homozygous for the same *TMPRSS3* variants. These were p.Ala138Glu (*n* = 6; eFigure 3D), p.Ala306Thr (*n* = 4; eFigure 3E), p.Val116Met (*n* = 1; eFigure 3F), and p.His70Thrfs*19 (*n* = 3; eFigure 3G).There were too few participants in each category for reliable statistical comparison.

### DFNB8 hearing loss progression

**Fig. 3:**
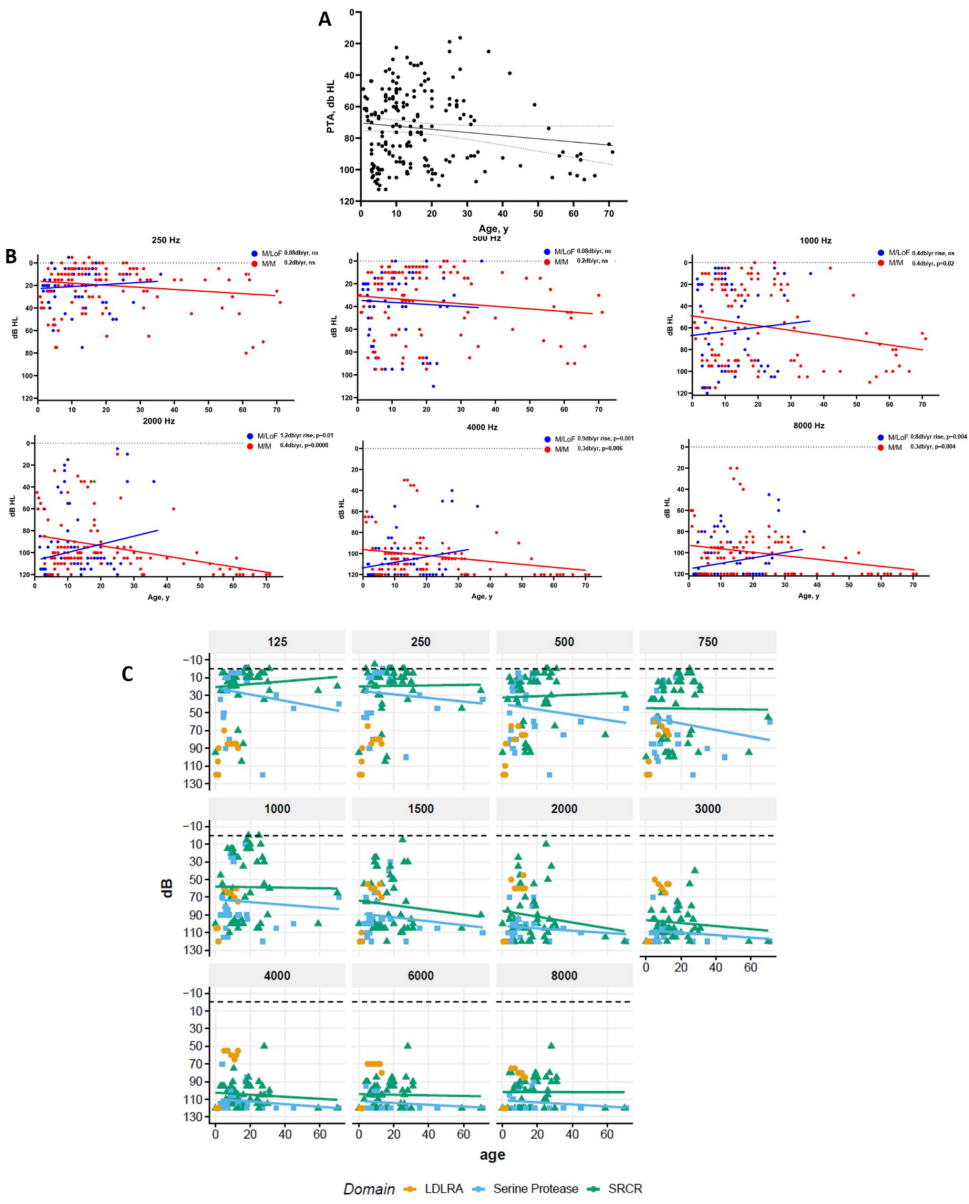
Hearing loss progression for individuals with DFNB8 genotype groups and protein domains. Pure tone averages (PTA) and hearing thresholds were plotted by age at test for individuals with DFNB8. **A** Changes in PTA by age were not significant (0.2db/year; *p* = 0.07 linear regression; dashed lines are 95% CI). **B** Hearing thresholds by frequency for each genotype group involved in DFNB8. Significant progression was seen for M/M genotypes at 1000 Hz, 2000 Hz, 4000 Hz, and 8000 Hz. **C** Progression was seen for individuals with two variants in the SRCR domain at 750–3000 Hz

When plotted by age, PTAs showed a hearing loss progression of 0.2 dB/year, but this was not significant (Fig. 3A; linear regression, 95% CI 0–0.4 dB/year, *R*^2^ = 0.02, *p* = 0.07). When hearing thresholds were analyzed by genotypic groups (Fig. 3B), significant progression was only seen for M/M individuals and only at 1000 Hz (0.4 dB/year, 95% CI 0.1–0.8 dB/year, *R*^2^ = 0.04, *p* = 0.019), 2000 Hz (0.4 dB/year, 95% CI 0.2–0.6 dB/year, *R*^2^ = 0.01, *p* = 0.0008), 4000 Hz (0.3 dB/year, 95% CI 0.1–0.5 dB/year, *R*^2^ = 0.07, *p* = 0.006), and 8000 Hz (0.3 dB/year, 95% CI 0.1–0.5 dB/year, *R*^2^ = 0.07, *p* = 0.004). Full statistics are available in eTable 8.

### Speech perception after cochlear implantation

**Fig. 4:**
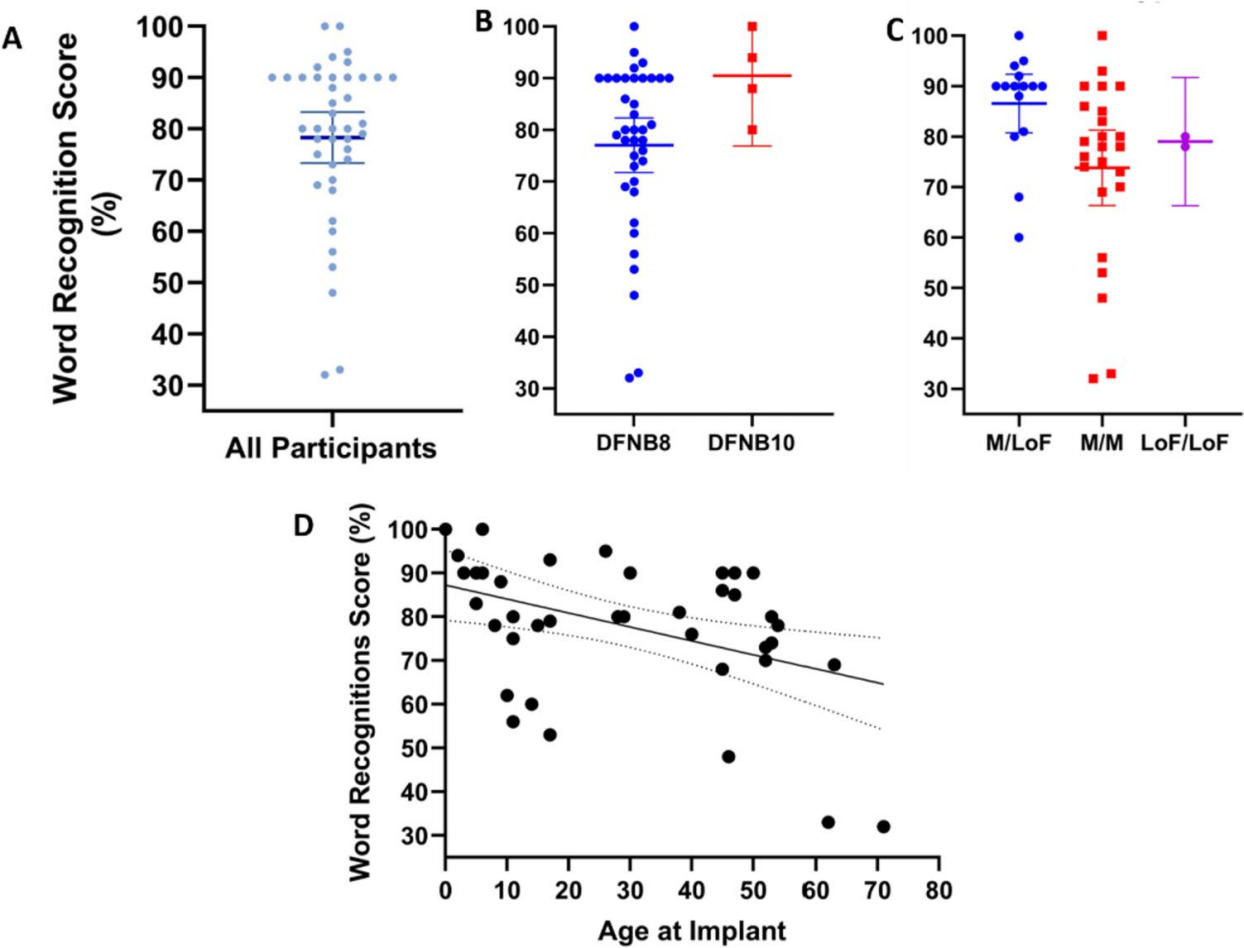
Speech perception score after cochlear implantation by DFNB8/10 and by genotypic categories. Word recognitions scores (WRS) were reported for 36 individuals. **A** The mean is 76% (95% CI 70–82%). Analysis by genotype (**B**) and DFNB8/10 (**C**) did not reveal any associations with WRS. **D** Worse WRS were associated with increased age at implantation (Dashed lines represent the 95% CI; Slope − 0.3190, *R*^2^ 0.1733, *p* = 0.0068, linear regression)

Seventy-four individuals in our data set had cochlear implants with 39 of these having word recognition scores (WRS) at least 1 year after implantation (Fig. 1A). The mean WRS for cochlear implant recipients with *TMPRSS3* variants was 76% (95% CI 70–82%; Fig. 4A). DFNB8, DFNB10, and the genotypic groups did not correlate with better or worse WRS (Fig. 4B, C). WRS score did correlate with age at implantation, with individuals who received their implant at older ages having a lower WRS (Fig. 4D; *p* = 0.007). Genotypes and age at implant of individuals who scored < 70% are available in eTable 9.

### TMPRSS3 protein modeling

**Fig. 5:**
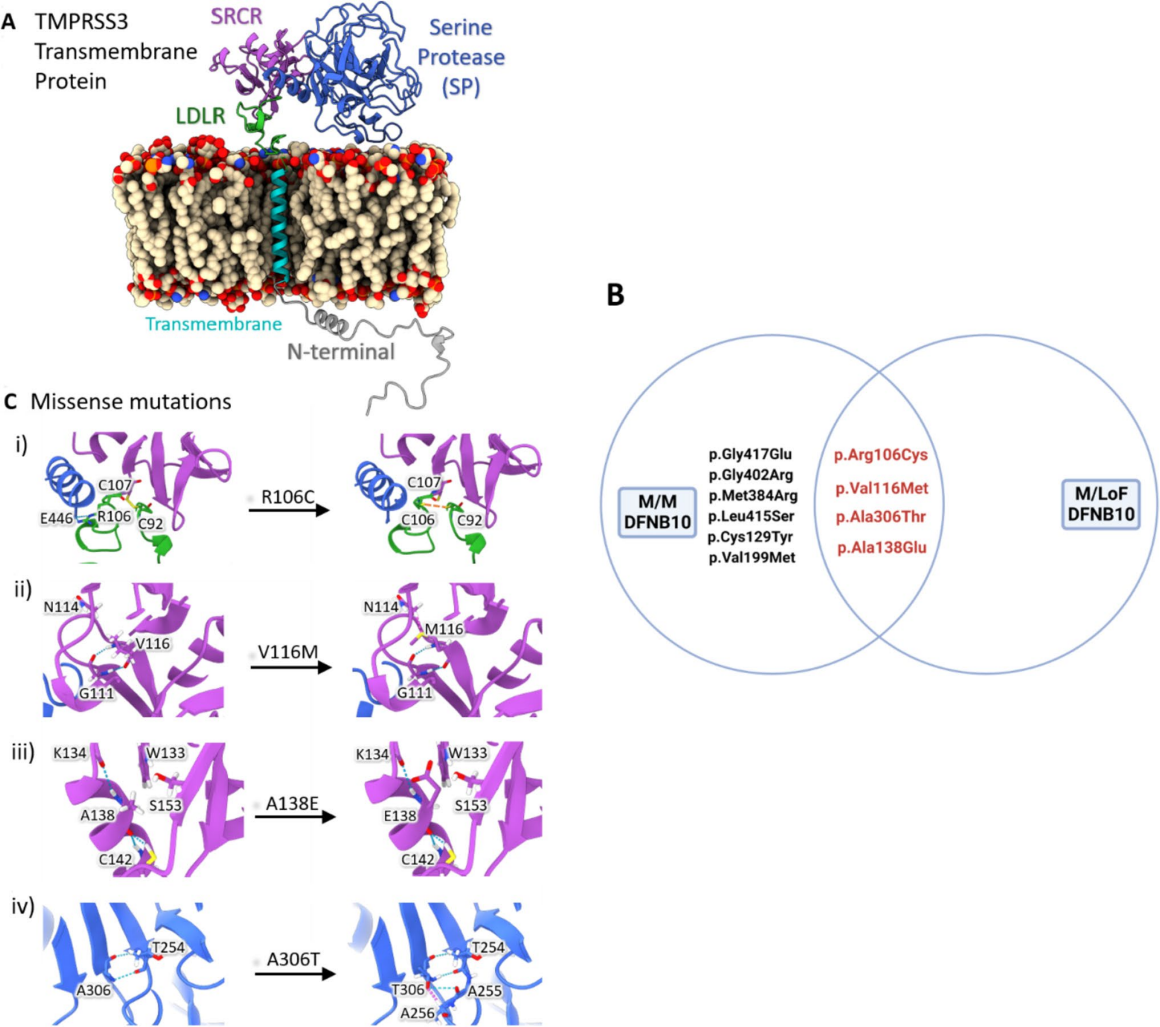
Protein modeling shows deleterious affects of 4 missense mutations. **A** Human TMPRSS3 model predicted by AlphaFold2, positioned in a lipid bilayer generated by Charmm-gui. The 4 domains of the protein are highlighted and labeled. **B** Overlap of missense variants that lead to severe hearing loss or M/WT with clinical hearing loss. The consistent overlap suggests these 4 variants are more severe in their effects. **C** Zoomed-in structures showing the differences in interactions due to these 4 missense mutations i) R106C mutation showing the potential disulfide bond formation in yellow dotted lines between C106 and C92. ii) V116M showing the clashing of the mutant Methionine with N114. iii) A138E shows the insertion of the large negatively charged Aspartate residue, inducing steric clashes with nearby amino acids W133, K134, and S153. iv) A306T showing two extra backbone hydrogen bonds formed by the mutant methionine residue with A255 and A256

The potential impact of all the *TMPRSS3* missense variants on protein structure and function was investigated (eTable 7). The 10 DFNB10 individuals with M/M genotypes (see ‘Audiometry Findings by DFNB and Genotypic Groups’ above) all had at least one allele of four missense variants: c.316C>T (p.Arg106Cys), c.346G>A (p.Val116Met), c.413C>A (p.Ala138Glu), or c.916G>A (p.Ala306Thr). This finding led us to hypothesize that these four variants significantly affected the protein structure and function, as they were associated with severe audiological phenotypes. Therefore, more extensive structural modeling was performed on these four variants (Fig. 5).

Structural modeling of the p.Arg106Cys variant in the LDLRA domain showed that this substitution is predicted to alter the structural conformation of three domains (LDLRA, SRCR and the serine protease) due to the unique location of this missense variant in a flexible loop at the interface between all three domains. Structural changes may be induced by the formation of a new disulfide bond between Cys92 and Cys106 instead of the nearby Cys107 leading to significant conformational changes (Fig. 5Ci). The two SRCR domain missense variants—Val116Met (Fig. 5Cii) and Ala138Glu (Fig. 5Ciii)—both appear to alter SRCR interactions, especially with a change from a hydrophobic residue to a negatively charged glutamate at position 138. The p.Ala306Thr (Fig. 5Civ) variant is located near the catalytic site of the serine-protease domain and is predicted to have a profound effect on the serine protease activity of TMPRSS3 by altering the structural configuration of the catalytic triad.

## Discussion

Variations in *TMPRSS3* are an important cause of genetic hearing loss (Guipponi et al. 2002). However, investigating the genotype–phenotype correlations of TMPRSS3 variants with hearing loss has been challenging due to the relatively small number of cases seen in most centers. This has resulted in vastly different conclusions about the natural history of TMPRSS3 hearing loss and cochlear implant outcomes (Carlson et al. 2023; Cremers et al. 1987; Eppsteiner et al. 2012; Lee et al. 2023; Shearer et al. 2017; Weegerink et al. 2011). To address these gaps in knowledge, we conducted a study with 127 individuals, which, to our knowledge, is the largest dataset on TMPRSS3 hearing loss. The aim of the study was to investigate the correlations between various genotypic categories and audiological phenotypes, hearing loss progression, and cochlear implant outcomes. This information is crucial for current clinical care and future therapeutic development.

### Variants are widely distributed across TMPRSS3 and are associated with varying severity

A total of 47 unique variants were identified across the *TMPRSS3* gene, indicating their widespread distribution throughout the gene and its protein domains (Fig. 1B, eTable 6). Notably, the serine protease domain, responsible for the protein's catalytic activity, harbored the majority (68%) of these variants. Among these variants, missense mutations occurring at the beginning of the serine protease domain were more frequently associated with the less severe DFNB8 (56 subjects), while variants located towards the end of the domain were associated with DFNB10 (Fig. 1B) in 22 subjects. While multiple variants across the whole gene were associated with only DFNB8 or DFNB10, nine variants were associated with both DFNB8 and 10 (Fig. 1B, eTable 4). This suggests there is more complexity to *TMPRSS3* phenotypes than the dichotomy of DFNB8 vs DFNB10, and this complexity may be driven by the specific combination of variants an individual possesses.

For example, certain variants such as p.Ala138Glu and p.Cys129Tyr were found to be associated with both DFNB types, but when combined with p.Ala306Thr, they specifically contributed to DFNB10. On the other hand, the c.323-6G>A splice site mutation and p.His70Thrfs*19 early termination variant were associated with DFNB10 when paired with another LoF allele, but when paired with a M allele, they were associated with DFNB8. Additionally, it was observed that some individuals with the same genotypes exhibited different levels of phenotypic severity, highlighting the significance of specific variant combinations in determining the resulting phenotype (eTable 4). To fully comprehend the complexity of these genotype–phenotype relationships, further analysis using larger datasets is necessary to adequately represent genotypes and consider other contributing factors that may influence the effects of these variants, such as modifiers, or gene–environment interactions.

As expected, greater phenotypic severity is seen with LoF variants; however, four M variants are associated with greater severity: p.Ala306Thr, p.Ala138Glu, p.Val116Met, and p.Arg106Cys (Fig. 5B). At least one of these variants are seen in each M/M individual who presented with profound DFNB10. Two of these four variants also overlap with missense variants that were observed in M/LoF individuals with profound DFNB10 (p.Ala306Thr and p.Ala138Glu; Fig. 5B). Protein modeling suggests these variants have severe effects on protein structure and function (Fig. 5C, Table S2). p.Arg106Cys is present in a site that interacts with three of the protein domains, LDLR, SRCR, and the serine protease. This variant is predicted to cause major protein misfolding and contributes to the severe hearing loss in M/M DFNB10 individuals. p.Ala306Thr is immediately adjacent to the catalytic region of the serine protease and likely interferes with its normal cleavage processes; it also likely contributes to the phenotypes of M/M DFNB10. p.Ala306Thr had also been previously hypothesized to lead to a severe phenotype (Weegerink et al. 2011) which was confirmed by a recent study (Lee et al. 2023) and our data (Fig. 5).

### Differences in TMPRSS3 phenotypes by genotype categories and protein domains

Overall, M/M and M/LoF genotypes presented with DFNB8 based on their audiogram profiles (Fig. 2D, E) and age at diagnosis (eFigure 2B). When DFNB10 individuals were subdivided by their genotype categories, we saw that all LoF/LoF individuals were DFNB10. These LoF/LoF-DFNB10 individuals show profound hearing loss (Fig. 2F) and an average age at diagnosis of 0.1 years (eFigure 2B). M/M, M/LoF, and LoF/LoF genotype groups only differ in thresholds at low frequencies (Fig. 2H). There were no significant differences between the genotypic categories at the middle and high frequencies. This lack of differences in hearing thresholds seen in our data set may be due to some M/M (*n* = 10) and M/LoF (*n* = 7) individuals displaying congenital, profound hearing loss, characteristic of DFNB10. Individuals with two variants in both the serine protease and SRCR domains have sloping audiograms (eFigure 3A, B), however, variants in the serine protease domain have higher thresholds at low frequencies than those with variants in the SRCR domain (47.5 dB HL vs. 32.5 dB HL), although this difference is not statistically significant (*p* = 0.36). Variants in the serine protease domain are associated with higher thresholds.

We investigated the impact of having two variants in the same domains (LDLRA, SRCR, or Serine Protease; *n* = 4, *n* = 14, and *n* = 17 subjects, respectively) and age at audiological test, on the thresholds for the different frequency bands (low, mid, and high), as well as the interactions of protein domain with frequency and age on the thresholds using linear mixed models (LMMs). LMMs are well suited for examining repeated measures and capturing shared variance within subjects while accounting for between-subject differences. This analysis revealed that the model explained 64% of the total variance, indicating a substantial contribution to the shape and severity of the audiogram by the domain affected. Furthermore, a highly significant association was observed between the threshold and frequency (shape of the audiogram) (*F*(2, 209) = 142.9, *p* < 2.2e−16) showing a steep-sloping hearing loss with large differences in thresholds between the low and high frequencies between the domains. This is particularly the case at low frequencies (250 and 500 Hz), where the LDLRA domain exhibited significantly worse thresholds compared to the other two domains; the differences between each domain were less pronounced at mid and high frequencies (eFigure 3A,B,C).

Additionally, a significant overall effect of the domain on the threshold was found (*F*(2, 32.1) = 7.2, *p* = 0.002622). Post hoc tests confirmed significant differences between the domains (serine protease vs. LDLRA *p* = 0.02; SRCR vs. LDLRA *p* = 0.002; SRCR vs. serine protease *p* = 0.4; eFigure 3A,B,C). In contrast, age at audiological test (*F*(1, 218) = 0.35, *p* = 0.56) and the interaction between domain and age (*F*(2, 71.1) = 1.3, *p* = 0.27) did not yield significant effects.

These findings indicate that variants in *TMPRSS3* primarily lead to high-frequency hearing loss, with the affected domain potentially contributing to hearing loss at mid- and low frequencies, especially for mutations in the LDLRA domain, and to a lesser extent for the SRCR and Serine Protease domains.

### DFNB8 hearing loss progression

One of the critical measures examined in this natural history study of *TMPRSS3* hearing loss is the quantification of hearing loss progression. A clear understanding of progression is essential to determine the therapeutic window for the treatment of DFNB8 and to provide accurate genetic counseling.

Since individuals with DFNB10 have profound, congenital hearing loss, we restricted our analysis of progression to individuals with DFNB8. Previous studies found that individuals diagnosed with DFNB8 progress at 3–10 dB/year (Carlson et al. 2023; Weegerink et al. 2011). In a previous study, M/M individuals showed statistically significant progression at 500 and 8000 Hz (3.5 dB/year and 0.9 dB/year, respectively), and M/LoF showed progression at all frequencies of 0.6–6 dB/year, depending on the frequency, with higher frequencies showing greater effects (Carlson et al. 2023). In our study, pure tone average (PTA) progression was 0.2 dB/year and was not statistically significant (Fig. 3A; 95% CI 0–0.4 dB/year, *p* = 0.07). M/M individuals showed statistically significant progression of 0.4 dB/year at 1000 Hz and 0.3 dB/year at 2000–8000 Hz (Fig. 3B). M/LoF individuals do not show statistically significant progression (Fig. 3B), possibly due to having higher thresholds than M/M at younger ages and across all frequencies. Alternatively, it is highly likely that specific combinations of M alleles with LoF alleles had differing effect strengths. The differences in hearing loss progression results between our study and previous studies may be accounted for by a larger sample size (69 DFNB8 individuals that had audiogram reports) with more *TMPRSS3* variants represented.

There were too few individuals homozygous for any one variant to make statistically significant associations between any one variant and progression. While some individuals did exhibit rapid progression, many individuals in our study had high hearing thresholds at young ages, leading to less pronounced progression findings for the population as a whole compared to previous studies.

### Cochlear implant outcomes

Cochlear implant outcomes for *TMPRSS3* have been widely debated, and data have pointed to both poor outcomes (Eppsteiner et al. 2012; Shearer et al. 2017; Tropitzsch et al. 2018) and good outcomes (Carlson et al. 2023; Chen et al. 2022; Lee et al. 2023; Moon et al. 2021; Tucker et al. 2021; Weegerink et al. 2011). The limitations of the previous studies were sample size. Our study had 36 individuals with cochlear implants and word recognition scores.

Speech perception tests vary by country and language which must be taken as a caveat to our findings. We found that individuals with *TMPRSS3* variants perform well on speech perception testing after cochlear implantation, with an average of 76% words correct (Fig. 4A; 95% CI 70–82%). We do not find a difference in outcomes by DFNB8/10 or by genotype groups (Fig. 4B,C) which is consistent with a recent study (Lee et al. 2023).

A previous study suggested that individuals with p.Ala138Glu were associated with poorer implant performance (Tucker et al. 2021). We found that individuals who performed worse than 70% had one of the following variants: p.Ala138Glu, p.Ala306Thr, p.Val116Met, p.Val199Met, or p.His70Thrfs*19 (eTable 9). However, our sample size was still too small to find statistically significant associations between specific variants and performance on word recognition tests.

We were able to show that older age at implant was associated with worse word recognition scores (Fig. 4D; *p* = 0.007) consistent with previous studies (Lee et al. 2023; Tucker et al. 2021). It remains unclear whether this observation is primarily attributed to the common correlations between older age and implant outcomes (Tamati et al. 2022), or if it is specifically related to the effects of living with a *TMPRSS3* variant for an extended period. Additionally, the duration of hearing aid usage before cochlear implantation could be another factor influencing the outcomes. Further study is necessary to properly investigate these possible explanations of poorer performance with age at cochlear implant.

### Limitations

Despite being the most extensive cohort study on TMPRSS3 conducted thus far, this study encountered challenges due to the high number of individual variants, many of which were compound heterozygotes in the participants. Consequently, establishing correlations between specific variants and distinct phenotypes proved to be difficult. Furthermore, the data collection process lacked standardization across individuals and centers. For example, some centers reported age at hearing loss onset and others age at exam and diagnosis; some reported serial audiological testing, while others had only one or no test reported. Additionally, the speech perception tests utilized in assessing cochlear implant outcomes varied by country and language. To gain a more comprehensive understanding, it is imperative to conduct an analysis that compares *TMPRSS3* implant outcomes with outcomes from variants in other well-known genes associated with hearing loss.

## Conclusions

Understanding the natural history of hearing loss-related variants is critical to determine how and when to intervene therapeutically (Nisenbaum et al. 2023; Pei et al. 2022). To successfully treat hearing loss, therapeutic advances must be accompanied by rapid progress in our understanding of the ever-expanding set of genotype–phenotype relationships. *TMPRSS3* is an important cause of genetic hearing loss. Previous studies were all limited by sample size. Although this study has a larger sample size (127 individuals in 115 families) to investigate the natural history of *TMPRSS3* variants and genotype-to-phenotype correlations, there remain limitations to this current data set. We found that there are differences in age of hearing loss diagnosis and audiological profiles by genotypic categories and which protein domain was affected. DFNB8 hearing loss progression is primarily seen for M/M individuals and variants in the SRCR domain. Cochlear implant outcomes are good, however, poor implant performance is seen and is driven by age at implantation with some evidence suggesting specific variants may play a role. Finally, four missense variants were associated with more severe phenotypes and protein structure changes. These findings provide insight for clinical care, genetic counseling, and therapeutic development (Du et al. 2023; Pei et al. 2022). On the path of therapeutic development, there is a greater therapeutic window for those with two missense alleles, so long as they are not one of the more severe variants identified. As we continue to build larger cohorts of individuals with *TMPRSS3*-related hearing loss, we will increase our predictive power and our ability to understand the therapeutic window and best approaches for the treatment of individuals with *TMPRSS3* hearing loss.

### Supplementary Information

Below is the link to the electronic supplementary material.

Supplementary file1 (PNG 551 KB)

Supplementary file2 (PNG 205 KB)

Supplementary file3 (PNG 705 KB)

Supplementary file4 (XLSX 75 KB)

## Data Availability

The datasets generated during and/or analyzed during the current study are available from the corresponding author on reasonable request.
