# Supplementary figures and images for: The natural history and genotype–phenotype correlations of TMPRSS3 hearing loss: an international, multi-center, cohort analysis
Source: Hum Genet. 2024 Apr 30;143(5):721–34. doi: 10.1007/s00439-024-02648-3 (PMC11098735; doi:10.1007/s00439-024-02648-3)

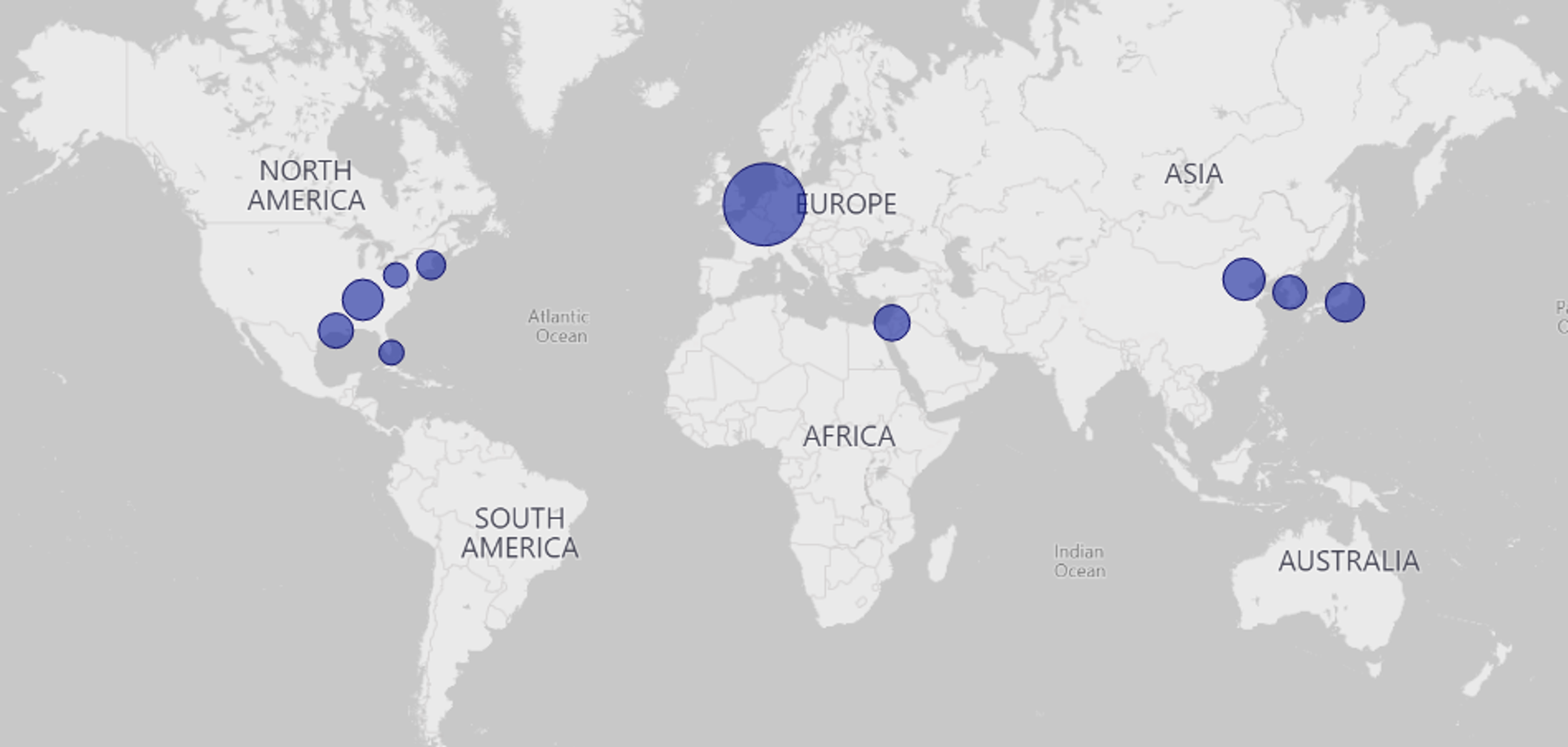

Supplement: Supplementary file 1 — Supplementary file1 (PNG 551 KB) [file 439_2024_2648_MOESM1_ESM.png]

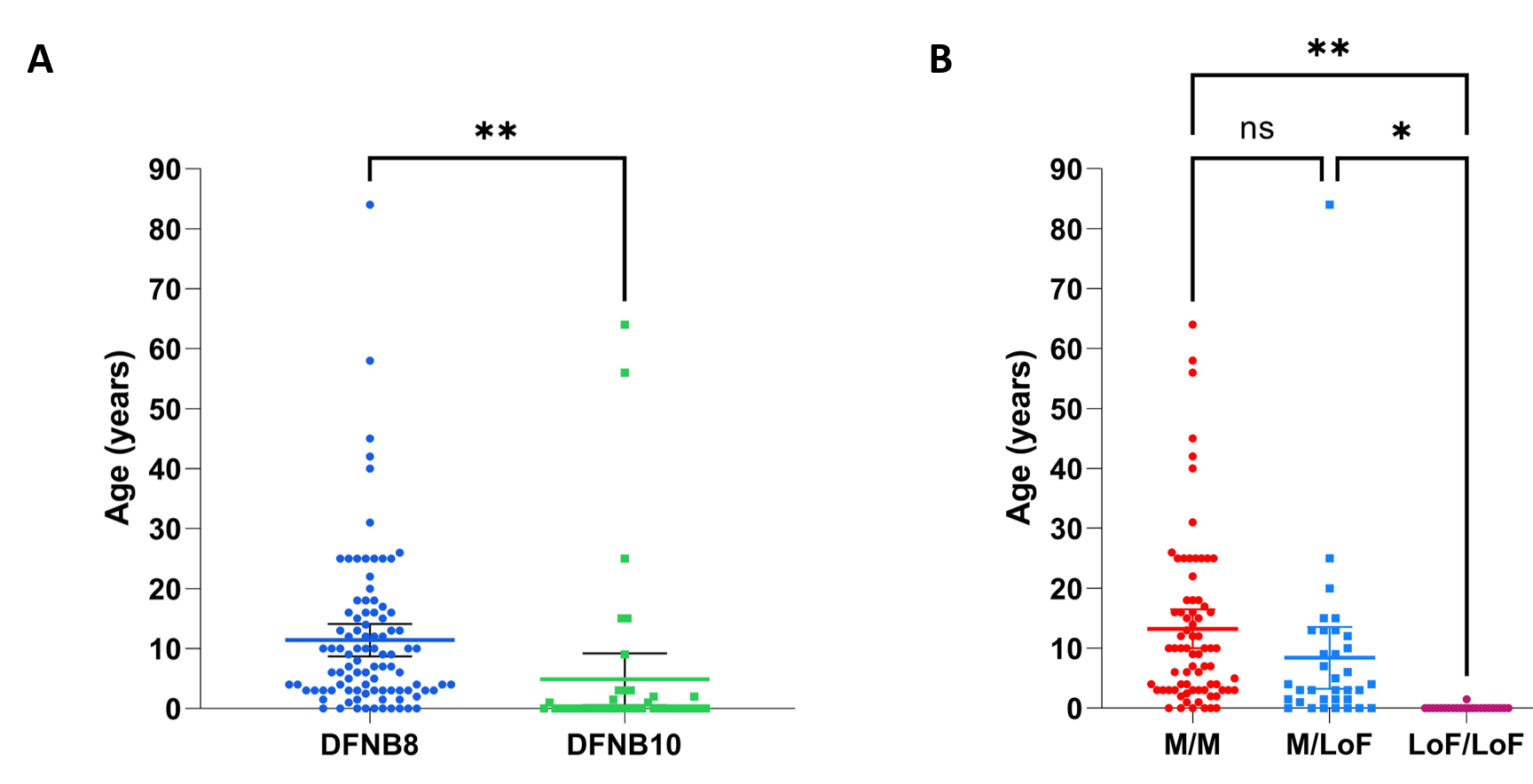

Supplement: Supplementary file 2 — Supplementary file2 (PNG 205 KB) [file 439_2024_2648_MOESM2_ESM.png]

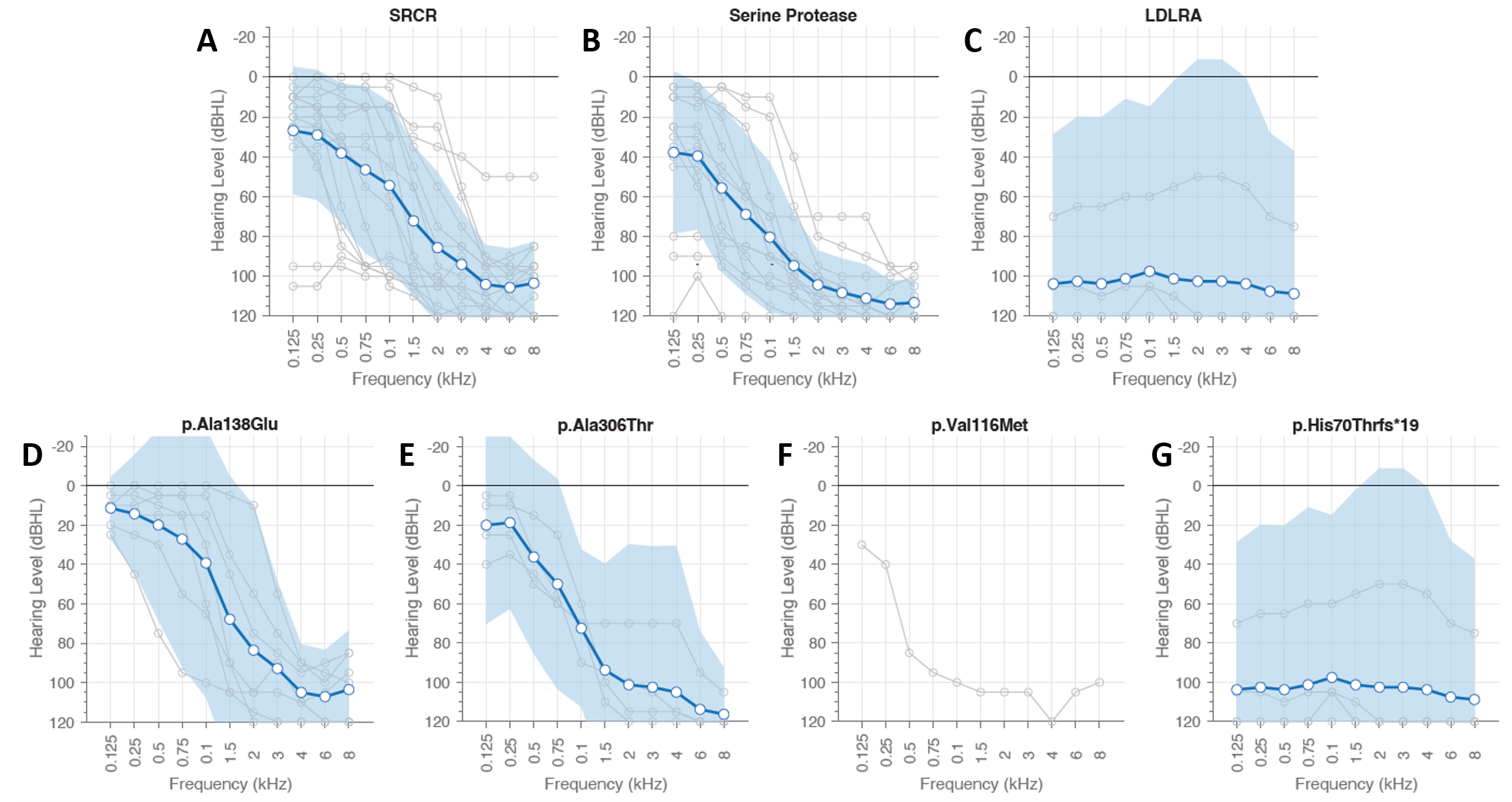

Supplement: Supplementary file 3 — Supplementary file3 (PNG 705 KB) [file 439_2024_2648_MOESM3_ESM.png]
